# Posttraumatic Symptoms and Posttraumatic Growth of Israeli Firefighters, at One Month following the Carmel Fire Disaster

**DOI:** 10.1155/2013/274121

**Published:** 2012-12-25

**Authors:** Dmitry Leykin, Mooli Lahad, Nira Bonneh

**Affiliations:** ^1^The Community Stress Prevention Centre, 11016 Kiryat Shmona, Israel; ^2^Department of Psychology, Tel Hai Academic College, 12210 Kiryat Shmona, Israel

## Abstract

Wildfire disasters are potentially traumatic events which directly and indirectly affect both citizens and first responders. The study of posttraumatic growth is scarcely found in the context of firefighters and only few studies have addressed this construct. In the current study, posttraumatic symptoms and posttraumatic growth were investigated among Israeli firefighters (*N* = 65), approximately one month after the Carmel Fire Disaster. Eight firefighters (12.3%) were found to be above the cut-off score for probable PTSD, with intrusion symptoms as the most frequent finding compared to avoidance and hyper-arousal symptoms. Posttraumatic growth (PTG) was evident to a small but considerable degree; noticeable changes were found regarding personal strength and appreciation of life. Results also revealed significant linear and quadratic relationships between PTSD and PTG. Results are discussed in light of past research on psychological responses among firefighters and first responders.

## 1. Introduction

At the end of December 2010, wildfires devastated large areas of the Carmel Mountain in northern Israel, burning almost 12,250 hectares of forest and an estimated 5 million trees. Two hundred and fifty homes and buildings were partially burnt; 74 were burnt to ashes. In the course of five days, roughly 25,000 people were evacuated from their homes as well as the occupants of one prison, one hospital, and one military jail. The fire, titled as the deadliest wildfire in the history of Israel, claimed the lives of 44 people, including prison service cadets, their commanding officers, two professional firefighters, and another volunteer firefighter (http://www.jnf.org/about-jnf/news/press-releases/after-the-fire.html).

Wildfire disasters, like other natural disasters, are potentially traumatic events which directly and indirectly affect both citizens and first responders [1, 2]. Evidence for PTSD rates among firefighters after fire disasters or bush fires is relatively scarce within the literature. Previous studies estimating PTSD prevalence have varied in assessment measurements and methodology. For instance, in a longitudinal design, McFarlane [3] examined firefighters exposed to Australian bushfires using the Impact Event Scale [4], but failed to report estimation of PTSD prevalence. Later, McFarlance and Papay [5] suggested that 42 months postbushfires, firefighters had 13% rate of PTSD. Bryant and Harvey [6] used the IES to examine posttraumatic stress reactions among firefighters exposed to bushfires and found that 9% presented extreme posttraumatic stress. More recently, Psarros et al. [7] investigated the psychosocial consequences of the August 2007 wildfires in Greece among professional firefighters and found that 18.6% of their sample suffered from PTSD, according to the criteria of the International Classification of Diseases (ICD-10). Other studies of firefighters' mental health following rescue and recovery efforts suggest probable PTSD rates ranging from 8.6% to 21% at different assessment times [8–11]. Due to the above, it is suggested that firefighters exposed to the Carmel fire disaster will demonstrate clinically significant PTSD rates of at least 10% one month after disaster.

The study of posttraumatic growth (PTG) [12], or “the constellation of positive changes that people may experience following a trauma or other stressful event” [13, p. 769], is scarcely found in the context of firefighters following natural disasters and only few studies have addressed this issue in such population [14, 15]. One such study, carried out by Gray [14], examined Canadian emergency service providers (78% of his sample were firefighters) who reported experiencing recent (up to 1 month) potentially traumatic events and found that traumatic stress was positively and significantly associated with posttraumatic growth (*r* = 0.41). Gray's interpretation of his results lays in the theoretical proposition that exposure to highly stressful or traumatic event is a necessary precondition for growth (Tedeschi and Calhoun, 1995). Melerski [15] interviewed thirty rescue workers (firefighters, police officers, and emergency medical technicians) who responded to the events of September 11th, 2001. In her sample, 87% of the participants indicated at least one positive outcome of the events, describing changes in appreciation of life, personal growth, and finding the experience of camaraderie to be positive. Other positive outcomes of the September 11th mentioned were focus on relationships that got closer, recognition of work, professional growth, better preparedness, changes in spirituality, and getting different life perspective. Earlier reports by Moran and Colless (1995) suggest that following certain emergency incidents, firefighters often describe sense of exhilaration, a sense of a job well done, a sense of appreciation of life and colleagues, and a sense of control over life′s vicissitudes. The authors failed to find any other study except Gary's work that addressed the association between posttraumatic symptoms and posttraumatic growth among firefighters [14]. Other studies, managed to examine the association between posttraumatic growth and posttraumatic symptoms among other first responders (e.g., ambulance drivers and police officers). For instance, Shakespeare-Finch et al. [16] explored the prevalence of self-reported positive changes among emergency ambulance personnel whose professional work demand high level of exposure to potentially traumatic experiences. Although this study concluded that occupational related trauma may act as a catalyst for significant positive posttrauma changes, it did not measure concurrent posttraumatic symptomatology and its relation to growth. In another study, Chopko [17] examined the relation between posttraumatic distress and posttraumatic growth among police officers following traumatic incidents. He showed that posttraumatic distress was significantly and positively related to the posttraumatic growth inventory full-scale and all subscale scores. 

The conclusions of some recent researche on PTG and distress are that growth is facilitated and maintained by endorsement rather than absence of post-traumatic symptomatology [18]. Additional body of evidence indicates curvilinear associations between growth and posttraumatic symptoms among traumatized individuals [19–21]. Since significant portion of these previous findings suggest a positive link between distress and growth among firefighters and first responders, it is predicted that both posttraumatic symptoms and growth symptoms will significantly correlate. The purpose of the current study is to expand current knowledge regarding both negative and positive reactions to traumatic events and their relationship among firefighters following a critical incident. 

## 2. Method

### 2.1. Participants

The participants (*N* = 65) of the present study were male firefighters (*M*  age = 36.64, SD = 7.93, range = 17–59) from a single district fire department. This fire department was among the first to respond to the blazing fire. Firefighters were formally contacted by a nongovernmental organization (NGO) that works with first responders during routine and crisis. Firefighters had a mean seniority of 13.35 years (SD = 7.63, range = 2–36 years) in their profession, represented more than 9 different roles in the fire department, over 80% were born in Israel, 96.9% were Jewish (60% were secular), 81% were married, 54% had a high school education, and the majority (50.0%) had an average income.

### 2.2. Measures

#### 2.2.1. Response to the Event

Seven responses to the event were measured using binary “yes” or “no” questions. Responses were to questions regarding injury to oneself, injury of others, perceived life threat, perceived threat to the life of others, feelings of helplessness, terror, and exposure to horrifying images at the scene. Questions were derived from the Posttraumatic Stress Diagnostic Scale (PDS; [22]).

#### 2.2.2. Impact of Event Scale-Revised (IES-R: [23])

The IES-R is a 22-item self-report questionnaire using a 5-point Likert scale, designed to measure the subjective response to a specific traumatic event in adults, measuring intrusion, avoidance, and hyperarousal symptoms, as well as a total subjective stress IES-R score, where higher scores indicate greater severity of posttraumatic distress. Participants were asked to indicate how distressing each difficulty (e.g., “I had trouble staying asleep” or “I thought about it when I did not mean to”) has been for them during the preceding seven days with respect to the fire disaster events. There is no specific cut-off score, although Creamer [24] reported a total score of 33 (or mean of 1.5) to be diagnostically accurate when compared to the PTSD checklist (PCL; [25]). A less conservative cut-off total score of 30 (and a mean of 1.4) was proposed by Asukai et al. [26]. Current testing indicated excellent reliability (*α* = .96). In the present study we measured probable PTSD even though not a full month has passed since the event, which is essential for DSM-IV diagnosis for PTSD [27]. However, the diagnosis of acute stress disorder (ASD) appears to be a strong predictor of subsequent posttraumatic stress disorder [28]. 

#### 2.2.3. Posttraumatic Growth-Short Form (PTGI-SF; [29])

A 10-item short form of the Posttraumatic Growth Inventory (PTGI; [12]) was administered to measure the degree of positive changes experienced in the aftermath of a traumatic event. Participants were asked to indicate the degree to which certain change (e.g., “I established a new path for my life” or “I know I can handle difficulties”) occurred in their life as a result of the fire disaster. According to Tedeschi and Calhun (1995) no time reference is needed. The PTGI-SF consists of five subscales, which represent the proposed domains of positive changes after trauma [12]; questions are answered on a 6-point Likert scale (*0—I did not experience this change as a result of my crisis* to *4—I experience this change to a very great degree as a result of my crisis)*. Previous confirmatory factor analyses have demonstrated a five-factor structure for the PTGI-SF equivalent to that of the PTGI [29]. Current testing indicated good reliability (*α* = .91).

### 2.3. Procedure and Data Analysis

Data was collected three and half weeks after the fire, at the beginning of a structured psychological group debriefing given to six groups of firefighters. Participants provided signed informed consent and were informed about the purpose of the assessment. Out of the approximate 120 firefighters and 30 officers in the district, 65 were included in the final sample (59 firefighters and 6 officers and the recruitment rate was 43.3%). While firefighters were in general compliance to participate, several logistic difficulties (e.g., current shifts/absenteeism) placed constraints on data collection. Refusal rates were not collected. One sample *t*-tests were used to test whether PTSD and PTG were significantly different from zero. A within-subjects ANOVA with repeated measures along with multiple comparisons (using Bonferroni corrected *P*-values) were performed in order to examine differences among the subscales of both the IES-R and the PTGI-SF. Curvilinear and linear relationships between posttraumatic symptoms and posttraumatic growth were examined using curve estimation regression analysis.

## 3. Results

Four firefighters reported that they were injured during the fire (6.5%). 49.2% reported that they felt their life was not in danger (although data regarding active participation in the fire intervention efforts is missing), and 77.4% reported that they perceived threat to the life of others. 33.9% responded that they experienced feelings of helplessness during the event, 14.8% reported feeling terror, and 57.4% reported being exposed to horrifying images at the scene.

 The mean total score on the IES-R was 15.60 (SD = 14.79, range 0–54) and the mean IES-R score was 0.71 (SD = 0.67), which was statistically different from zero, *t*(64) = 8.52, *P* < .001. Eight firefighters (12.3%) were found to be above the conservative cutoff score for probable PTSD. According to the less conservative cut-off, twelve firefighters (18.5%) fulfilled the criteria for probable PTSD. When examining PTSD subscales (intrusion, avoidance, and hyper-arousal), the intrusion mean score was found to be significantly higher than the other two subscales, while no difference was found between avoidance and hyper-arousal, *F*(2, 128) = 20.57, *P* < .001.

 The mean total score on the PTGI-SF was 20.61 (SD = 12.49, range 0–43) and the mean score was 2.08 (SD = 1.27), which was statistically different from zero, *t*(64) = 12.77, *P* < .001. When comparing the five components of posttraumatic growth, it was found that changes in personal strength and appreciation of life were rated to a greater extent, *F*(4, 256) = 9.96, *P* < .001. Post hoc multiple comparisons indicated that changes in personal strength were significantly much greater than the other changes, except for changes in appreciation of life, which in turn were significantly greater than changes in the relation to others and new possibilities in life.  Table 1 presents the means and standard deviations of PTSD symptoms and posttraumatic growth.

**Figure 1:**
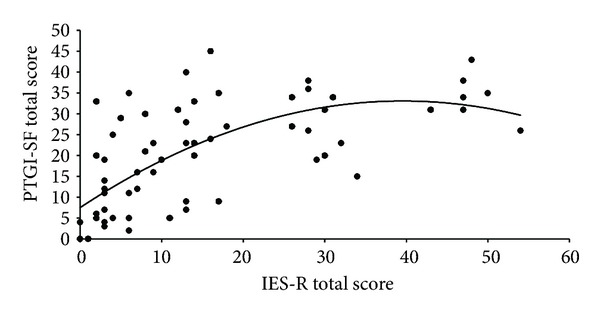
Curvilinear relationship between posttraumatic symptoms and posttraumatic growth.

 In order to examine the possibility of a curvilinear and linear relationship between posttraumatic symptoms and posttraumatic growth, curve estimation regression analysis of trauma symptom levels was carried. Results revealed significant linear and quadratic relationships for the total PTG score (linear *R* ^2^ = .396, *b*_1_ = 1.20, *F*(1, 63) = 41.23, *P* < .001; quadratic *R* ^2^ = .465, *b*_1_ = 2.84, *b*_2_ = −.77), *F*(2,62) = 26.95, *P* < .001) (see Figure 1). In other words, besides the linear relations (as posttraumatic symptoms increase, and growth increases as well and vice versa) and a curvilinear (inverted U) relationship between PTSD symptoms and posttraumatic growth was found, that is, participants who reported intermediate levels of symptoms experienced higher levels of growth than those reporting low or high levels of symptoms. A test of significance between the two correlation coefficients indicated that the curvilinear relationship was not significantly stronger than the linear relationship between posttraumatic distress and growth, *Z* = −.47, n.s.

Posttraumatic growth symptoms were associated with a severity response index (summed index of event responses), *r*(62) = .22, *P* = .092, although this relationship reached marginal significance. More specifically, changes in relatedness to others were significantly associated with severity of response to the event index, *r*(58) = .31, *P* < .05. The severity of the response to the event was positively and statistically significant correlated with posttraumatic symptoms, *r*(62) = .28, *P* < .05. Neither posttraumatic symptoms nor growth were associated with family status, income, seniority, and education.

## 4. Discussion

The present study indicates that, overall, firefighters were significantly mentally affected by the fire disaster. This finding resembles previous studies showing that first responders, like nonprofessional responders who are exposed to large scale disasters, experience mental health difficulties [7]. However, the current probable PTSD prevalence and IES-R was found to be lower than the estimates for firefighters responding to hurricanes and earthquakes, reported in a review by Neria et al. [2]. The PTSD rates which were estimated in the review at 2 and 5 months after disaster were higher than the current study's rates, possibly due to the delayed onset of PTSD observed in firefighters following natural disasters [30]. However, when comparing findings regarding acute onset of PTSD rates of firefighters responding to fire disasters [30], current rates of PTSD were not so different than previously reported (9.2%). Estimation of delayed onset of PTSD was not part of the current study, thus the course of PTSD over time could not be evaluated. Intrusion symptoms were the most salient cluster of PTSD symptoms experienced by the firefighters, resembling prior findings of symptom manifestations following terror attacks [31] and earthquakes [32] among first responders. 

Though feelings of helplessness during the event is an essential feature of Criterion A1 for ASD and PTSD [27], it has not been systematically investigated. In the current study, more than one-third of the firefighters reported feelings of helplessness during the fire disaster, a considerably higher rate than the ones that have been previously reported [6, 33]. 

In the current study, the positive outcomes of the fire disaster reported by the firefighters suggest that following a disaster, first responders, albeit small but still to a significant degree, were able to experience positive changes, especially in their perceived personal strength and appreciation of life. These findings fit with qualitative reports of firefighters indicating at least one positive outcome of the September 11th events, describing changes in their appreciation of life and personal growth [15]. 

A significant relationship between the severity of posttraumatic symptoms and posttraumatic growth emerged from the findings, suggesting a possible correlation between both salutary and pathological outcomes [14, 34]. In accordance with past research [20, 34], a significant curvilinear relationship between growth and posttraumatic symptoms emerged, indicating there may be an optimal level of posttraumatic distress that promotes growth. The results, however, do not suggest that growth and distress association is better interpreted by curvilinear relationship. A limitation of the current study relates to the small sample size, lack of information regarding perceived organizational support, and other organizationally related variables which have previously been found to correlate with both PTSD symptoms and growth [14]. As noted before, observing the pattern of posttraumatic symptoms and concluding regarding probable PTSD before a full one month had passed is another limitation to the present study. It is noteworthy that during this time period, majority of people who experience posttraumatic stress reactions in the initial weeks after trauma show reduction of symptoms in the following months [35]. As much as the authors support the notion that assessment of PTSD needs to adhere to the official guidelines, in the current population the three and half weeks assessment could have been an indicator to follow those exhibiting higher PTS and maybe start an intervention to reduce PTSD rates, as a qualitative follow-up report indicates in this specific incident [36]. Finally, since most of the cited studies in the present paper investigated firefighters' psychopathology following man-made disasters, more emphasis should be directed in future to the psychological consequences (both the negative and the positive) of natural disasters upon firefighters.

## Figures and Tables

**Table 1:** Means and *t* scores for posttraumatic symptoms and posttraumatic growth (*N* = 65).

Variable	*M*	SD	*t* ^ a^
IES-R^b^	0.71	0.67	8.52***
Intrusion	0.87	0.75	9.35***
Avoidance	0.67	0.71	7.56***
Hyperarousal	0.56	0.66	6.81***
PTGI-SF^c^	2.04	1.29	12.77***
Relating to others	1.64	1.16	11.35***
New possibilities	1.62	1.33	9.80***
Personal strength	2.32	1.57	11.89***
Spiritual growth	1.75	1.62	8.71***
Appreciation of life	2.08	1.53	10.99***

^a^One Sample *t*-test, different from zero;

^b^IES-R: Impact of Event Scale-Revised (0–4);

^c^Posttraumatic Growth Inventory-Short Form (0–5); df = 64.^∗^ *P* < .05*;* ^∗∗^ *P* < .01; (two-tailed).
